# Ex Vivo Modeling and Pharmacological Modulation of Tissue Immune Responses in Inflammatory Bowel Disease Using Precision‐Cut Intestinal Slices

**DOI:** 10.1002/eji.70013

**Published:** 2025-07-24

**Authors:** Klaudia Maria Grieger, Valerie Schröder, Susann Dehmel, Vanessa Neuhaus, Dirk Schaudien, Maximillian Fuchs, Helena Linge, Alexander Wagner, Ulf Kulik, Benjamin Gundert, Heiko Aselmann, Armin Braun, Christina Hesse, Katherina Sewald

**Affiliations:** ^1^ Fraunhofer Institute for Toxicology and Experimental Medicine Hannover Germany; ^2^ Member of the German Center for Lung Research (DZL) Biomedical Research in Endstage and Obstructive Lung Disease Hannover (BREATH) Research Network Hannover Germany; ^3^ Member of the Fraunhofer Excellence Cluster of Immune Mediated Diseases (CIMD) Frankfurt am Main Germany; ^4^ Institute of Molecular and Translational Therapeutic Strategies Hannover Medical School (MHH) Hannover Germany; ^5^ Department For General Visceral and Transplantation Surgery Hannover Medical School (MHH) Hannover Germany; ^6^ KRH Klinikum Region Hannover (Germany) Clinic for General Visceral and Minimally Invasive Surgery Hannover Germany

**Keywords:** immune modulation, inflammatory bowel disease, intestinal immunity, Precision‐cut intestinal slices (PCIS), Th17

## Abstract

Inflammatory bowel disease (IBD) affects approximately 5 million people worldwide, causing chronic inflammation and increased mortality. Despite advances in therapy, the underlying immune mechanisms remain poorly understood, highlighting the need for human‐immunocompetent models to enhance translational research. This study aimed to investigate local immune responses using precision‐cut intestinal slices (PCIS) from IBD patients and evaluate immunomodulatory treatment directly in patient tissue ex vivo. PCIS from ileal resections of IBD and non‐IBD patients were stimulated with Concanavalin A (ConA) or lipopolysaccharide (LPS). Histological analysis of IBD‐derived PCIS showed villus atrophy, infiltration of lymphocytes and macrophages, and RNA analysis revealed upregulation of IL‐17 and interferon signaling pathways. LPS‐ and ConA‐induced functional immune responses in the tissue, with IBD tissue exhibiting increased levels of specific cytokines compared with non‐IBD tissue, including IL‐17F and IL‐21 after ConA‐stimulation, and IL‐22 as well as ENA‐78 following LPS‐stimulation. Pimecrolimus treatment led to a marked reduction in the release of IL‐2, IL‐17A, and IFN‐γ, and inhibited the IBD supernatant‐induced reduction in transepithelial electrical resistance. Our data provide the first in‐depth characterization of local tissue immune responses in human PCIS, highlighting the potential of this model to study disease‐specific immune activity and evaluate pharmacological interventions ex vivo.

## Graphical abstract

Studying local immune responses in IBD remains challenging. This study demonstrates that precision‐cut intestinal slices from IBD patients mimic key immunopathological features ex vivo and respond specifically to immune stimuli. This model allows direct pharmacological modulation of patient tissue ex vivo and offers a powerful platform for translational research.

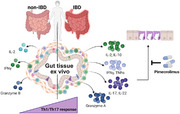

**Abbreviations**
ATP: adenosine triphosphate
CD: cluster of differentiation
ConA: concanavalin A
DAPI: 4′,6‐diamidino‐2‐phenylindole
ENA‐78: epithelial neutrophil‐activating protein 78
GM‐CSF: granulocyte‐monocyte colony‐stimulating factor
IBD: inflammatory bowel disease
IFN: interferon
IL: interleukin
LDH: lactate dehydrogenase
LPS: lipopolysaccharide
NFAT: nuclear factor of activated T cells
PCIS: precision‐cut intestinal slices
Pim: Pimecrolimus
TEER: transepithelial electrical resistance
Th: T helper
TNF: tumor necrosis factor
TRAIL: tumor necrosis factor‐related apoptosis‐inducing ligand

## 1 Introduction

Inflammatory bowel diseases (IBD) are lifelong, progressive disorders characterized by chronic recurrent inflammation of the intestinal mucosa. IBD include Crohn's disease, which can affect any segment of the gastrointestinal tract, and ulcerative colitis, which is primarily located in the colon [1]. Common symptoms of IBD include abdominal pain, diarrhea, rectal bleeding, and weight loss [2]. Additionally, IBD represents a high‐risk factor for other diseases such as colorectal cancer, cardiovascular and respiratory disorders, leading to higher mortality rates in these patients compared with the healthy population [3, 4]. Approximately 5 million people worldwide suffer from IBD, with prevalence steadily increasing [5]. IBD typically begins in early adulthood and progresses in a chronic, relapsing pattern, representing an immense burden on healthcare and socioeconomics [1, 6]. Despite current advances in research, the etiology and pathogenesis of IBD are still not fully understood.

Clinical and experimental studies indicate that in genetically susceptible individuals, the disease is driven by an abnormal immune response to environmental triggers, leading to damage of the intestinal barrier, tissue injury, and persistent inflammation [7, 8]. The inflammatory phenotype in IBD is characterized by a massive infiltration of Th1 and Th17 cells into the gut, along with increased serum levels of cytokines such as IL‐17, IL‐21, IFN‐γ, and TNF‐α [9, 10]. In addition, an increased frequency of cytotoxic CD8^+^ T cells has been observed in IBD [11, 12]. Proinflammatory mediators such as IFN‐γ, TNF‐α, as well as perforin and granzymes released by cytotoxic T cells, potentially exacerbate tissue damage by acting directly on the intestinal epithelial barrier [11, 13].

Recent advances in IBD therapy came from the development of biologicals, targeting proinflammatory cytokines or their receptors involved in the disease pathology [14, 15]. Standard therapy for IBD patients consists of corticosteroid treatment. If this approach fails, biologicals as well as other anti‐inflammatory drugs, for example, JAK/STAT inhibitors or calcineurin inhibitors, are applied [16, 17, 18]. However, the proportion of nonresponders (30–50%) and patients who become refractory to treatment (10%) remains high [15]. Among these alternatives, calcineurin inhibitors offer a targeted approach to modulate T cell activity in diseased tissues. Calcineurin, a Ca^2+^‐calmodulin‐dependent serine/threonine phosphatase, plays a key role in T cell differentiation, proliferation, and activation. Upon activation, calcineurin dephosphorylates the nuclear factor of activated T cells (NFAT), facilitating its translocation to the nucleus, where it promotes the transcription of key T cell genes like IL‐2 and CD25 [19, 20].

The development of new therapies for IBD requires advanced human‐relevant models that reflect the complexity of the intestinal microenvironment. Organoid‐based systems have provided important insights into epithelial biology and disease mechanisms [21]. While immune cells can be included, these models typically contain a selectively introduced repertoire of immune cell types, which may partly reflect the diversity and spatial organization of cells in native tissue [22]. Microphysiological systems such as gut‐on‐a‐chip models offer additional features, including flow dynamics and the integration of selected immune components, thereby increasing physiological relevance [23, 24]. Nevertheless, they are technically complex and often rely on a limited number of predefined cell types. Intestinal biopsy cultures preserve native tissue architecture and cellular heterogeneity, but factors such as limited nutrient diffusion can affect viability in deeper layers and may impact reproducibility, especially for longer incubations [25, 26]. Ussing chambers, which are widely used for transport studies, require comparatively large tissue samples and are typically limited to short‐term experiments [27, 28].

Precision‐cut intestinal slices (PCIS) offer an alternative that combines native tissue architecture with standardized handling. They preserve the full cellular complexity of the intestinal wall, including immune cells present in the patient's tissue, and allow multiple conditions and replicates to be tested in parallel from a small biopsy sample [29, 30, 31]. Their reproducibility and extended viability make them a promising tool for studying complex cellular responses in IBD. Human PCIS are ultrathin, viable tissue sections that have been used for drug metabolism, transport, and toxicology studies as well as in research on food allergies [32, 33, 34]. In this study, for the first time, we comprehensively analyzed local immune responses in primary tissue slices from patients with and without inflammatory bowel disease. To assess the possibility of pharmacological intervention in PCIS, we treated tissue slices ex vivo with the calcineurin inhibitor pimecrolimus. We hypothesized that pimecrolimus reduces T cell activity, in particular, increased Th17 cell‐associated responses, in primary intestinal tissue from IBD patients ex vivo. Additionally, we propose that reducing the release of T cell mediators would rescue the intestinal barrier damage, typically observed in IBD patients.

To test this, we used viable intestinal tissue slices from ileum resections of IBD and non‐IBD patients as well as an intestinal epithelial barrier model. Our data show increased release of several disease‐relevant mediators in primary intestinal tissue from IBD patients, which can be inhibited by ex vivo treatment with pimecrolimus. Further, our study demonstrates that the increased activity of T cells in IBD tissue not only contributes to inflammation but also plays a key role in epithelial barrier damage that can be addressed by T cell‐targeted therapeutic approaches, such as pimecrolimus.

## 2 Results

### 2.1 Primary Intestinal Tissue of IBD Patients Resembled Pathogenic Innate and Adaptive Immune Signatures Ex Vivo

**FIGURE 1:**
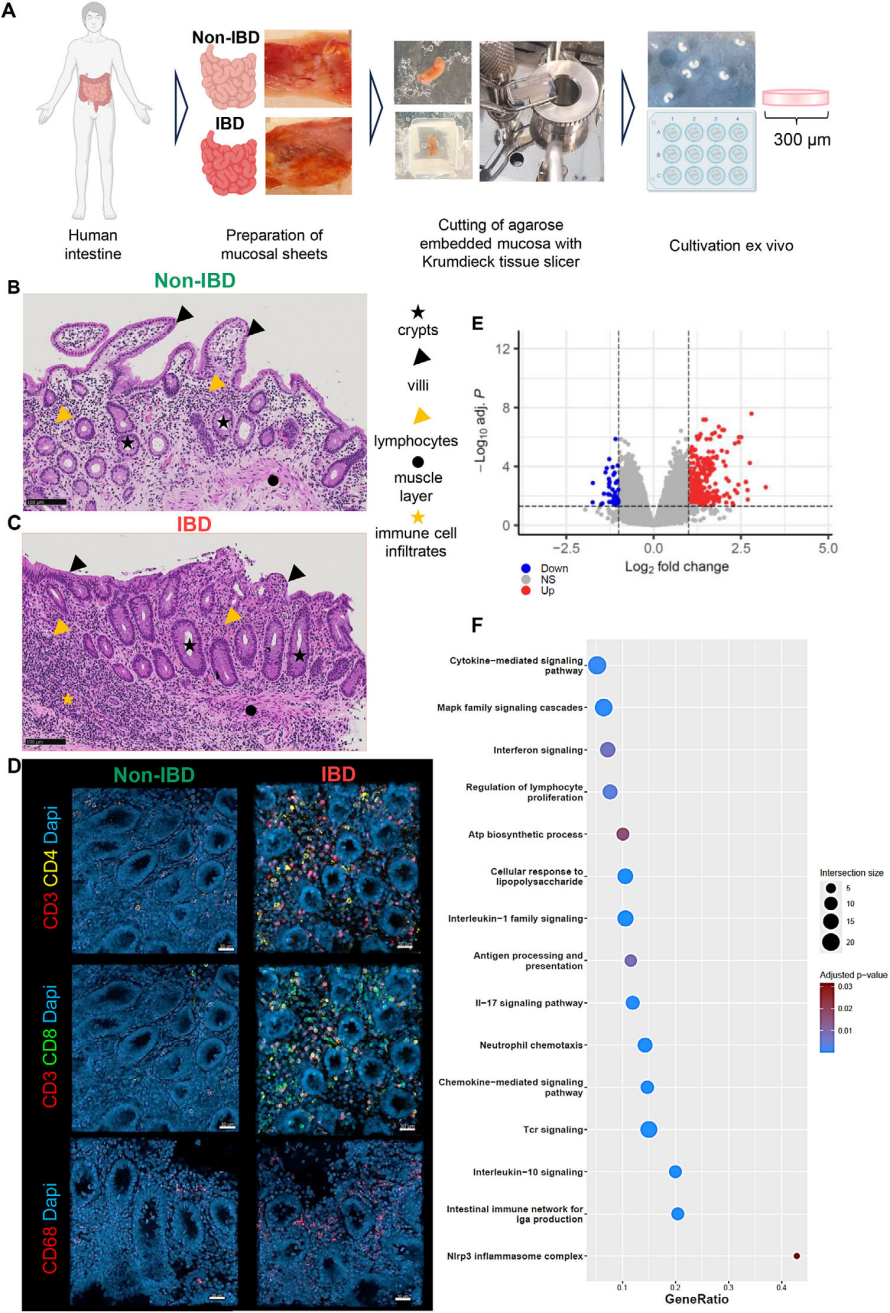
Primary intestinal tissue slices prepared from patients with IBD and cultured ex vivo maintained disease‐typical morphology and biomarker secretion. Schematic representation of tissue slice preparation and cultivation **(A)**. Hematoxylin & eosin staining of formalin‐fixed, paraffin‐embedded thin sections (4 µM) of non‐IBD **(B)** and IBD **(C)** tissue slices after 24 h cultivation, exemplary intestinal structures and cell types are labelled with geometric shapes, representative images of PCIS from *N* = 4 IBD and non‐IBD donors each. Immunofluorescence images of whole‐mount paraformaldehyde‐fixed non‐IBD and IBD **(D)** tissue slices. Tissue slices were stained for CD3 or CD68 (red: AlexaFluor 647), CD4 (yellow: AlexaFluor 568), CD8 (green: AlexaFluor 488), and DAPI (blue). Panels showing CD3/CD4/DAPI and CD3/CD8/DAPI represent the same tissue sections stained for different markers. Representative images of *N* = 3 IBD and non‐IBD donors each. Upregulation of gene signature between PCIS from IBD and non‐IBD patients was analyzed using Clariom S microarray. **(E)** Volcano plot showing Log2 fold‐changes relative to adjusted p values of genes in ileal PCIS samples, *N* = 5 donors (IBD‐derived PCIS) and *N* = 4 donors (non‐IBD‐derived PCIS), per donor two technical replicates (two wells with two tissue slices each). NS (grey): not significant, up (red): significantly higher in IBD‐ vs. non‐IBD‐derived PCIS, down (blue): significantly lower in non‐IBD‐ vs. IBD‐derived PCIS. **(F)** Bubble plot of selected significantly enriched biological pathways and processes in IBD‐ vs. non‐IBD‐derived PCIS obtained from over‐representation analysis. (A) was created with BioRender.com.

Viable primary intestinal tissue slices were prepared from terminal ileum resections of patients with Crohn's disease (IBD) or tumor‐free terminal ileum resections of patients with other background diseases, for example, colon carcinoma (non‐IBD) (Figure 1A, Table 1). Cellular composition, gene expression, and biomarker secretion were characterized in intestinal tissue slices of both IBD and non‐IBD patients. Primary intestinal tissue slices from non‐IBD patients exhibited intestinal‐typical morphology, characterized by circular crypts and villi with a columnar epithelial monolayer containing Paneth cells with eosinophilic granules and goblet cells with basophilic mucin‐filled vacuoles. The lamina propria displayed a normal cellular composition, including lymphocytes, granulocytes, and other immune cells, and muscularis mucosae under the lamina propria with cigar‐shaped muscle cells (Figure 1B; Figure S1B). In contrast, IBD tissue exhibited pathological changes such disordered epithelial cell layer and increased cellularity in the lamina propria, with dense clusters of lymphocyte infiltrates (Figure 1C; Figure S1C). Analysis of the lymphocyte composition in primary intestinal tissue slices from IBD patients revealed increased presence of CD4^+^ and CD8^+^ T cells in the subepithelial layer, compared with tissue from non‐IBD patients (Figure 1D). We also found an increased presence of CD68+ macrophages in the subepithelial layers of IBD tissue slices compared with non‐IBD tissue slices (Figure 1D). Analysis of gene expression using microarray technology revealed 407 differentially expressed genes with 364 up‐ and 43 downregulated genes in tissues of IBD compared with non‐IBD patients, respectively (Figure 1E; Table S1). Of note, overrepresentation analysis (ORA) using differentially expressed genes showed several upregulated pathways of the innate and adaptive immune response, including response to lipopolysaccharide, T cell receptor signaling, IL‐17 signaling, interferon signaling, and lymphocyte proliferation in IBD tissue (Figure 1F; Table S2). In addition, the biosynthetic capacity for ATP was found to be upregulated at the genetic level in IBD tissue slices compared with non‐IBD tissue slices, likely reflecting increased metabolic activity of cells in the tissue (Figure 1F).

**TABLE 1:** List of human intestine donor material used in this study.

Donor No.	Sex	Age	Disease background	Surgical details	Medical treatment	Used for
1	M	44	Crohn's disease	Ileocecal resection in case of ileitis terminalis, stenosis and interenteric fistulas	Adalimumab	Ex vivo PCIS stimulation + gene expression, PCIS‐SN on C2BBe1
2	W	58	Rectal adenoma	Ileostomy reversal	No known Tx	Ex vivo PCIS stimulation + gene expression
3	M	42	Crohn's disease	Ileocecal resection in case of entro‐cutaneous fistula	Infliximab	Ex vivo PCIS stimulation + gene expression, PCIS‐SN on C2BBe1
4	M	46	Appendix carcinoma	Hemicolectomy with ileotransversostomy	No known Tx	Ex vivo PCIS stimulation + gene expression, PCIS‐SN on C2BBe1
5	M	59	Sigma diverticulitis	Ileostomy reversal	No known Tx	Ex vivo PCIS stimulation, PCIS‐SN on C2BBe1
6	M	44	Crohn's disease	Residual colectomy and discontinuity resection in case of interenteric fistula	No known Tx	Ex vivo PCIS stimulation + gene expression, PCIS‐SN on C2BBe1
7	W	52	Rectal adenoma	Ileostomy reversal	No known Tx	Ex vivo PCIS stimulation PCIS‐SN on C2BBe1
8	M	68	Rectal adenoma	Ileostomy reversal	No known Tx	Ex vivo PCIS gene expression
9	M	72	Colon carcinoma	Ileostomy reversal	No known Tx	Ex vivo PCIS stimulation + gene expression, PCIS‐SN on C2BBe1
10	W	64	Sigma diverticulitis	Ileostomy reversal	No known Tx	PCIS‐SN on C2BBe1
11	W	36	Crohn's disease	Ileostomy reversal in case of stenosis	Azathioprin, Infliximab, Mesalazine, Adalimumab	Ex vivo PCIS gene expression, PCIS‐SN on C2BBe1
12	W	29	Crohn's disease	Proctocolectomy with placement of a terminal ileostomy in case of ileocolitis with fistula	Azathioprin+Allopurinol, UstekinumabVedolizumab	Ex vivo PCIS gene expression

*Note*: All tissue samples were obtained from the terminal ileum. Characteristics of donors, including disease background, surgical details, medical treatment, and the experiments that were performed with this donor.

Abbreviations: M: male, W: female, Tx: treatment, SN: supernatant.

**FIGURE 2:**
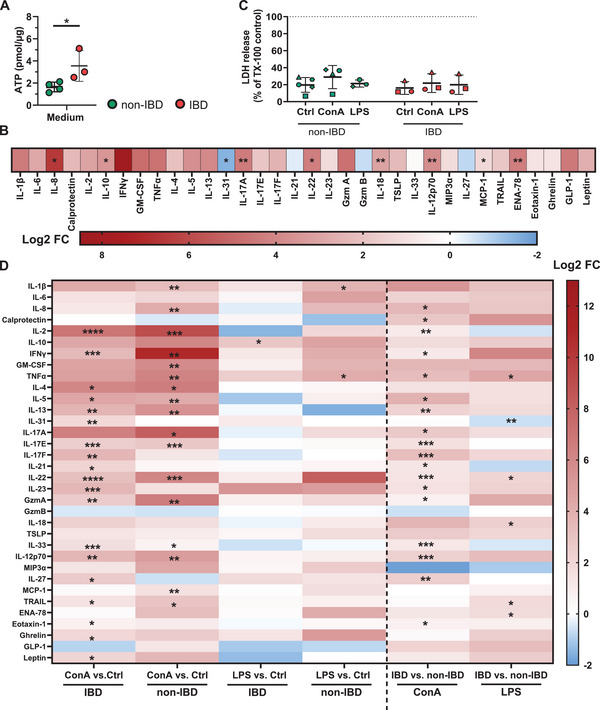
Concanavalin A and bacterial lipopolysaccharide induced distinct pathogenic innate and adaptive immune signatures in IBD tissue ex vivo. For viability measurement, LDH and ATP assays were performed. Tissue slices were cultivated for 24 h. **(A)** Intracellular ATP levels normalized to µg total protein of the tissue. Each dot represents one donor, *N* = 3–4. Per donor, a minimum of two technical replicates (two wells with two tissue slices each) were analyzed. *p < 0.05 by unpaired, two‐tailed t‐test (non‐IBD vs. IBD). **(B)** Unstimulated tissue slices were cultivated in medium (Ctrl) for 24 h to determine baseline secretion of several mediators in supernatant of IBD versus non‐IBD tissue slices, presented as Log2 normalized fold changes (log2FC). *N* = 3–4 donors (two technical replicates per donor) were analyzed. *p < 0.05, **p < 0.01 by unpaired two‐tailed *t*‐test between IBD and non‐IBD. Tissue slices were stimulated with 10 µg/mL Concanavalin A (ConA) or 1 µg/mL lipopolysaccharide from E. coli O111:B4 (LPS) for 24 h, and the absolute secretion of several mediators in supernatant, as well as viability, was analyzed. **(C)** LDH release in supernatant normalized to LDH release of Triton X‐100 lysed control tissue slices. **(D)** Absolute secretion of several mediators. Log2 normalized fold changes (log2FC) of secreted mediator levels in ConA‐stimulated (ConA) vs. unstimulated (ctrl) PCIS from IBD or non‐IBD patients and LPS‐stimulated (LPS) vs. unstimulated (ctrl) PCIS from IBD or non‐IBD patients (left). Log2FC of secreted mediator levels in PCIS from IBD vs. non‐IBD patients after ConA or LPS stimulation (left). *N* = 3–4 non‐IBD and *N* = 3 IBD donors. Per donor, a minimum of two technical replicates (two wells with two tissue slices each) were analyzed. *p < 0.05, **p < 0.01, ***p < 0.001, ****p < 0.0001 by unpaired two‐tailed *t*‐test comparing either ctrl vs. ConA or ctrl vs. LPS (right) or IBD vs. non‐IBD (left).

To comprehensively analyze functional immune responses in the tissue, we first confirmed the viability of intestinal tissue slices over 24 h in culture. Metabolic activity, as measured by intracellular ATP levels, was maintained in tissues from both IBD and non‐IBD patients (Figure S1A). However, consistent with the gene expression analysis, tissue slices from IBD patients displayed elevated levels of ATP compared with tissue slices from non‐IBD patients (Figure 2A). To unravel functional differences in the immune status of tissues, we initially assessed the baseline secretion of immune mediators after 24 h in culture (Figure 2B, Table S3). Tissue slices from IBD patients showed increased baseline secretion of several proinflammatory mediators, including the Th17‐related cytokines IL‐17A (log2FC: 4.38), IL‐22 (log2FC: 4.24), and Th1‐related cytokine IL‐12p70 (log2FC: 3.75) as well as several mediators associated with the recruitment of innate immune cells, such as IL‐8 (log2FC: 6.59), MCP‐1 (log2FC: 1.26) and ENA‐78 (log2FC: 4.27). Notably, secretion of the clinically relevant biomarker calprotectin was increased in IBD‐derived PCIS already at baseline (log2FC: 2.32), although this did not reach statistical significance. In contrast, IBD tissue showed, compared with non‐IBD tissue, lower baseline secretion of IL‐31 (log2FC: −1.54), IL‐21 (log2FC: −0.38), granzyme B (log2FC: −0.71), and IL‐27 (log2FC: −0.79), reaching statistical significance for IL‐31 (Figure 2B; Table S3).

To characterize functional responses of tissue‐resident immune cells and determine disease‐specific mediator secretion in more detail, we stimulated primary intestinal tissue slices with the antigen‐independent T cell mitogen Concanavalin A (ConA) or bacterial lipopolysaccharide (LPS) as an innate immune stimulus. Both ConA and LPS triggered the secretion of immune mediators in IBD‐ and non‐IBD‐derived PCIS without affecting the viability of the tissue (Figure 2C,D). ConA showed overall more pronounced effects on cytokine secretion than LPS (Figure 2D; Tables S3 and S4). Compared with unstimulated controls, LPS upregulated the secretion of IL‐10 (log2FC: 2.8) in IBD tissue and IL‐1β (log2FC: 3.8) and TNF‐α (log2FC: 4.2) in non‐IBD tissue. LPS‐stimulated tissue from IBD patients showed, compared with LPS‐stimulated tissue from non‐IBD patients, increased secretion of mediators associated with the recruitment of innate immune cells and tissue repair, such as TNF‐α (log2FC: 4.4), IL‐22 (log2FC: 2.1), IL‐18 (log2FC: 2.4), TRAIL (log2FC: 1.85), and ENA‐78 (log2FC: 1.82), but, similar to unstimulated conditions, decreased secretion of IL‐31 (log2FC: −0.64).

Analysis of secreted mediator levels after ConA stimulation of PCIS revealed significant differences in the absolute ConA‐induced release between IBD and non‐IBD tissue, but also the reactivity of the tissue to ConA, indicating different cellular compositions and activation status (Figure 2D). Responsiveness to ConA compared with unstimulated controls was generally higher in tissue from non‐IBD patients than in tissue from IBD patients. In non‐IBD‐derived PCIS, ConA stimulation led to an increased secretion of general proinflammatory cytokines (e.g., IL‐1β, IL‐8, MCP‐1; log2FCs: 2.49–4.23), but also specific T cell‐associated cytokines (e.g., IL‐2, IFN‐γ, GM‐CSF, TNF‐α, IL‐17A, IL‐22, GzmA; log2FCs: 5.84–10.81) (Figure 2D). In IBD tissue, ConA mainly induced the release of T cell‐associated cytokines, including IL‐2, IFN‐γ, IL‐17F, IL‐17E, and IL‐22 (logFCs: 2.48–7.07). Notably, IL‐31 (log2FC: 1.9), IL‐17F (log2FC: 3.68), IL‐21 (log2FC: 2.17), IL‐23 (log2FC: 3.64), and IL‐27 (log2FC: 2.68) were upregulated only in tissue from IBD patients, but not in tissue from non‐IBD patients, after ConA stimulation (Figure 2D). Direct comparison of absolute mediator levels in ConA‐stimulated IBD compared with ConA‐stimulated non‐IBD tissue slices confirmed increased secretion of T cell‐associated cytokines, indicating an intensified Th1, Th17, but also cytotoxic T cell response in IBD tissue (Figure 2D). Further, levels of clinically relevant biomarkers IL‐8 and calprotectin were increased in ConA‐stimulated IBD tissue slices compared with ConA‐stimulated non‐IBD tissue slices (Figure 2D). Of note, the Th2‐related cytokines IL‐4, IL‐5, IL‐13, and IL‐33 were upregulated by ConA in tissue from both non‐IBD and IBD patients, with absolute levels of IL‐5, IL‐13, and IL‐33 being higher in tissue from IBD patients compared with non‐IBD patients. However, compared with Th1, Th17, and cytotoxic T cell‐associated mediators, the overall levels of these cytokines were relatively low (Figure 2D; Tables S3 and S4).

### 2.2 Pimecrolimus Inhibited Pathogenic T Cell Responses in Primary Intestinal Tissue of IBD Patients Ex Vivo

**FIGURE 3:**
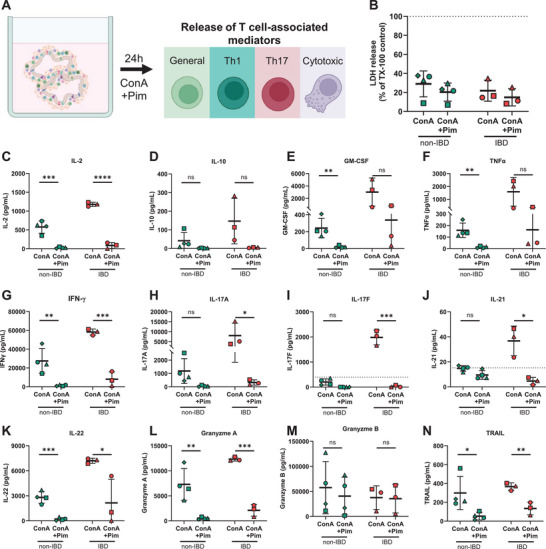
Pimecrolimus inhibited ConA‐induced mediator release in IBD and non‐IBD tissue ex vivo. Tissue slices were stimulated with 10 µg/mL Concanavalin A (ConA) ± 25 µM pimecrolimus (Pim) for 24 h, and the release of T cell‐associated mediators was analyzed, schematic representation **(A)**. LDH release in supernatant normalized to LDH release of Triton X‐100 lysed control slices **(B)**. IL‐2 **(C),** IL‐10 **(D)**, GM‐CSF **(E)**, TNFα **(F)**, IFN‐γ **(G)**, IL‐17A **(H)**, IL‐17F **(I)**, IL‐21 **(J)**, IL‐22 **(K)**, granzyme A **(L)**, granzyme B **(M),** and TRAIL **(N)** levels in supernatant. Each geometric shape represents an individual donor, non‐IBD: N = 4, IBD: *N* = 3. Per donor, a minimum of two technical replicates (two wells with two tissue slices each) were analyzed. *p < 0.05, **p < 0.01, ***p < 0.001, ****p < 0.0001, ns: not significant by unpaired two‐tailed *t*‐test comparing ConA vs. ConA+Pim. **(A)** was created with BioRender.com.

As a proof‐of‐concept for pharmacological modulation of local immune responses in primary intestinal tissue slices ex vivo, we tested intervention with pimecrolimus, focusing on the modulation of Th1‐, Th17‐, and cytotoxic T cell‐associated responses. Pimecrolimus is a calcineurin inhibitor known to suppress proinflammatory cytokine release and T cell function. In our experiments, we treated primary intestinal tissue slices with ConA and pimecrolimus simultaneously for 24 h. Subsequently, we examined the release of Th1‐, Th17‐, and cytotoxic T cell‐associated mediators ex vivo (Figure 3A). Of note, combined treatment with ConA and pimecrolimus had no cytotoxic effect on the tissue (Figure 3B). Pimecrolimus reduced the ConA‐induced release of IL‐2 in tissue from non‐IBD patients (22‐fold) and tissue from IBD patients (14‐fold), indicating efficient inhibition of T cell activation ex vivo (Figure 3C). Concomitantly, IL‐10 secretion was inhibited in both tissues (Figure 3D). Pimecrolimus further significantly decreased the ConA‐induced release of GM‐CSF (non‐IBD: 12‐fold; IBD: 9‐fold), TNF‐α (non‐IBD: 12‐fold; IBD: 10‐fold), IFN‐γ (non IBD: 21‐fold; IBD: 7‐fold), IL‐17A (non‐IBD: 24‐fold; IBD: 25‐fold), IL‐17F (not quantifiable in both tissues after treatment), IL‐21 (non‐IBD: 1.5‐fold; IBD: 8‐fold), and IL‐22 (non‐IBD: 13‐fold; IBD: 3‐fold), indicating inhibition of Th1‐ and Th17‐cell associated responses ex vivo (Figure 3E–K). Moreover, pimecrolimus reduced the ConA‐induced release of granzyme A and TRAIL in both tissues from IBD (granzyme A: 6‐fold, TRAIL: 2.6‐fold) and non‐IBD (granzyme A: 14‐fold, TRAIL: 6‐fold) patients (Figure 3L,N), whereas granzyme B secretion remained unaltered in both groups (Figure 3M). Among the ConA‐induced Th2‐associated cytokines, a significant reduction of IL‐33 (2‐fold) in tissue of non‐IBD patients, as well as significant reduction of IL‐4 (19‐fold), IL‐5 (12‐fold), IL‐13 (3‐fold), and IL‐33 (5.5‐fold) in tissue from IBD patients was observed (Figure S3).

Taken together, the results of our study showed that pimecrolimus had a significant effect on the release of multiple T cell‐associated mediators induced by ConA in tissue from both non‐IBD and IBD patients, demonstrating efficient inhibition of IBD‐related pathogenic T cell responses ex vivo.

### 2.3 Pimecrolimus Reduced the Release of Barrier‐Damaging Mediators From IBD Patient Tissue

**FIGURE 4:**
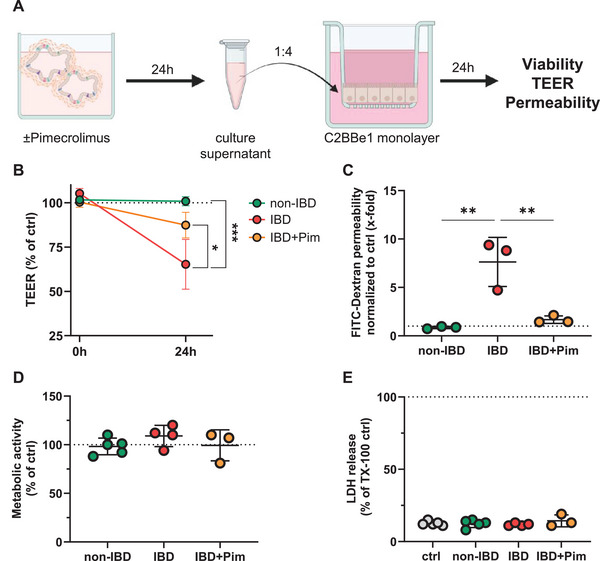
Treatment of IBD tissue with pimecrolimus reduced the release of barrier‐damaging soluble mediators. Supernatants of tissue from non‐IBD patients, tissue from IBD patients, or tissue from IBD patients treated with 25 µM pimecrolimus (Pim) were incubated on C2BBe1 monolayers for 24 h. Culture supernatants were mixed with C2BBe1 culture medium at a ratio of 1:4. A 1:4 mixture of tissue culture medium and C2BBe1 culture medium was included as medium control (Ctrl) **(A)**. Transepithelial electrical resistance (TEER) of C2BBe1 cells at 0 and 24 h, normalized to Ctrl at 0 h (dotted line, set to 100%) **(B)**. Permeability of C2BBe1 cells by FITC‐labeled dextran permeability assay at 24 h, measured fluorescence units were normalized to Ctrl (dotted line, set to 1) **(C)**. Metabolic activity of C2BBe1 cells by WST‐1 assay at 24 h, normalized to Ctrl (dotted line, set to 100%) **(D)**. LDH release in apical supernatants at 24 h, normalized to Triton X‐100 lysed control (dotted line, set to 100%). Each dot represents one individual donor, from which supernatants for incubation on C2BBe1 cells were collected. **(B)**, **(D),** and **(E)**: *N* = 5 donors (non‐IBD) and *N* = 3 donors (IBD and IBD+Pim); **(C)**: *N* = 3 donors (non‐IBD, IBD, and IBD+Pim). Per donor, one experimental run with two technical replicates was performed. *p < 0.05, **p < 0.01, ***p < 0.001 by one‐way ANOVA with Dunnett's multiple comparisons test (IBD vs. non‐IBD and IBD vs. IBD+Pim). **(A)** was created with BioRender.com.

To assess the barrier‐damaging effects of soluble mediators released from primary IBD tissue, we incubated intestinal epithelial cell monolayers (Caco‐2 clone C2BBe1) with conditioned supernatant (unstimulated or treated only with pimecrolimus) from primary intestinal tissue cultures of IBD and non‐IBD patients and assessed the transepithelial electrical resistance (TEER), cell permeability, and cell viability (Figure 4A). Pimecrolimus reduced baseline secretion of IL‐2, IL‐17A, and IL‐22 in tissue from IBD patients (Figures S2 and S3). Culture supernatant from the tissue of non‐IBD patients showed no effect on the barrier integrity of C2BBe1 cells. In contrast, culture supernatant from tissue of IBD patients reduced the TEER of C2BBe1 monolayers up to 35% and increased Fluorescein‐labeled dextran permeability approximately eightfold (Figure 4B,C). This effect was partially inhibited when IBD tissue was treated with pimecrolimus before incubation of supernatants on C2BBe1 cells (IBD vs. IBD+Pim: TEER: 65 ± 14% vs. 89 ± 9.5% of control and Fluorescein‐labeled dextran permeability: 8.4 ± 2.5‐fold vs. 2.2 ± 0.8‐fold of control), indicating that T cell‐released mediators were critical for the observed effects on barrier integrity (Figure 4B,C). Notably, tissue culture supernatants did not affect the overall viability of C2BBe1 cells (Figure 4D,E), underlining that the observed effects on barrier integrity were most likely direct effects on the epithelial junctional properties. Of note, adding pimecrolimus to IBD‐conditioned supernatants after PCIS culture did not have a detectable direct effect on the TEER or viability of C2BBe1 epithelial cells, further demonstrating that the previously observed effects were related to the action of pimecrolimus on tissue‐resident cells in PCIS rather than being direct effects of pimecrolimus on C2BBe1 cells (Figure S4).

## 3 Discussion

The development and progression of IBD is influenced by a complex interplay of genetic, epigenetic, environmental, and immunological factors, leading to an impaired intestinal barrier integrity and elevated T cell accumulation, particularly Th1/Th17 cells, which drive inflammation [9, 35]. Both Th1 and Th17 cells contribute to inflammation in IBD by inducing epithelial cell apoptosis and exacerbating mucosal injury [10].

Preclinical models used in IBD research are primarily based on in vivo mouse colitis models [36, 37]. In patients, analysis of tissue‐specific immune responses is often hampered by the limited availability of relevant material. However, studying these responses is essential as immune cells circulating in blood do not necessarily represent tissue‐resident populations. In line with this, other studies have shown that PCIS cultured *ex vivo* maintain tissue‐resident immune cells capable of reacting to exogenous stimuli [30, 38, 39].

However, the immune response of tissue‐resident T cells and disease‐related differences in local tissue immunity in human PCIS have not been studied so far. Our study aimed to characterize in detail functional innate and adaptive immune responses to relevant immunological mitogens in ex vivo primary intestinal tissue from IBD patients compared with non‐IBD patients and to investigate the immunomodulatory and barrier‐protective effects of a pharmacological intervention, pimecrolimus, targeting T cell activity.

Tissue slices from IBD patients exhibited IBD‐typical characteristics, including immune cell infiltrates and elevated secretion of known IBD‐related biomarkers, such as IL‐8, calprotectin, IL‐17A, and IFN‐γ, which were preserved in our ex vivo culture [40, 41, 42, 43]. Further, we observed an increased basal release of pro‐inflammatory cytokines, for example, IL‐22, IL‐12p70, and ENA‐78, aligning with the upregulation of genes involved in IL‐17 signaling, IFN signaling, and neutrophil chemotaxis in IBD‐derived PCIS ex vivo. This suggests persistent activation of proinflammatory cells, contributing to the chronic inflammation characteristic of the IBD‐derived PCIS microenvironment [44, 45]. The secretion of IL‐31 and IL‐27 was found to be downregulated in IBD as compared with non‐IBD tissue slices, possibly due to their roles in maintaining tissue homeostasis and regulating immune responses, which may be suppressed in the strong proinflammatory environment of IBD [46, 47]. Consistent with previous studies highlighting the importance of T cells and macrophages in IBD, PCIS from IBD patients showed increased accumulation of CD68+ cells as well as CD4^+^ and CD8^+^ T cells in the lamina propria [48, 49, 50, 51]. Further, IBD tissue showed, compared with non‐IBD tissue, increased intracellular ATP levels, indicating increased metabolic activity of cells within the tissue.

Activated T cells have been shown to increase aerobic glycolysis over mitochondrial oxidative phosphorylation for the production of ATP, as this results in an increased availability of important precursor molecules needed for the biosynthesis of nucleic acids, lipids, and amino acids [52, 53]. Acute changes in the metabolism of tissue‐resident cells in PCIS could be further explored by assessing the ATP:AMP ratio in the tissue, oxygen consumption, and/or extracellular acidification [54]. Stimulation of tissue slices with the antigen‐independent T cell mitogen ConA resulted in increased release of T cell‐associated mediators, including Th17, Th1‐ and cytotoxic T cell‐associated mediators, in tissue from IBD patients. In contrast, LPS stimulation of PCIS induced the secretion of key innate cytokines, including IL‐1β, TNF‐α, and IL‐22, particularly in non‐IBD tissue slices, while IBD tissue slices showed a weaker response. Bacterial LPS is primarily recognized by TLR‐4, which is expressed by mononuclear cells in the intestinal lamina propria and intestinal epithelial cells [55, 56]. One possible explanation is that intestinal tissue‐resident cells are constantly exposed to high levels of LPS and must regulate proinflammatory responses to this stimulus to maintain homeostasis. In IBD, an impaired epithelial barrier leads to increased exposure to luminal components, including bacterial metabolites [57], which might further enhance tolerance to LPS stimulation. However, absolute levels of secreted TNF‐α, IL‐22, IL‐18, TRAIL, and ENA‐78 were still higher in LPS‐stimulated IBD compared with LPS‐stimulated non‐IBD tissue slices, which is likely representative of an increased presence of proinflammatory tissue‐resident cells such as macrophages. ENA‐78, IL‐22, and IL‐18, released by macrophages or epithelial cells, are particularly important for promoting neutrophil chemotaxis, leading to increased inflammation and tissue damage [58, 59, 60].

Importantly, Th17 cells have been described to have a dual role in intestinal (patho‐)physiology. Dependent on the cytokine milieu, Th17 cells can either exert protective effects supporting mucosal barrier integrity or pathogenic effects contributing to chronic inflammation and tissue damage [35, 61, 62, 63]. Pathogenic Th17 cell responses have been directly implicated in exacerbating intestinal inflammation in IBD [9, 10]. In our study, ConA stimulation increased secretion of IL‐17F, IL‐21, IL‐22, and IL‐23 in primary IBD tissue slices, reflecting an enhanced pathogenic Th17 cell response ex vivo. Notably, IL‐22 was also inducible in non‐IBD tissue, although to lower levels, while IL‐17F, IL‐21, and IL‐23 were upregulated only in IBD tissue slices.

The role of IL‐22 and IL‐21 in the pathogenesis of IBD is still debated, as both cytokines have been described to exert protective effects, but also provoke inflammation in a context‐dependent manner [64, 65, 66]. Our study provides direct functional evidence for the differential regulation of these cytokines in human intestinal tissue, highlighting IL‐21 and IL‐17F as discriminating factors between tissue from IBD and non‐IBD patients. Further, we observed increased secretion of IFN‐γ, TNF‐α, and GM‐CSF in IBD tissue slices after ConA stimulation compared with non‐IBD tissue slices. IFN‐γ is a hallmark Th1 cytokine; however, mouse studies have shown that its release by Th17 cells contributes to the complexity of the inflammatory response associated with IBD. In this context, the IL‐12/IL‐23/STAT axis is relevant as it enhances the induction of both Th subtypes, a process closely associated with the development of colitogenic phenotypes [10, 35, 67]. Pathogenic Th17 cells are known to co‐produce IFN‐γ and IL‐17A and have been described to secrete increased levels of GM‐CSF and IL‐22 as well [10]. Upregulated expression of pathogenic Th17 cell‐related genes and cytokines, as well as increased abundance of pathogenic Th17 cells in the intestinal mucosa, has also been observed in IBD patients [68, 69, 70]. Our data provide direct functional evidence for the role and differential regulation of (pathogenic) Th17 cell‐associated mediators in IBD patient tissue.

Further, cytotoxic T cells contribute to IBD pathology by attacking the intestinal epithelial lining, causing tissue damage and exacerbating inflammation [11, 13]. Granzymes are proteases, which are released from T cell granules upon activation and implicated in this process [71]. Boschetti et al. [72] found elevated granzyme B‐ and perforin‐producing T cells in the blood and ileal mucosa of recurrent IBD patients, suggesting that they may play a role in the initiation of gut lesions. We observed elevated secretion of granzyme A, but not granzyme B, in tissue slices from IBD patients’ ex vivo. Granzyme A showed protective, antibacterial effects in the context of intestinal infections [73], and its increased secretion in IBD tissue slices may be related to increased exposure to intestinal pathogens due to intestinal epithelial barrier damage.

Multiple cytokines investigated in this study, including TNF‐α, IFN‐γ, and IL‐17A, have been shown in other studies to affect the epithelial barrier by causing degradation and relocalization of tight junction proteins such as occludin and zonulin‐1 [70, 74, 75]. In this study, we could demonstrate a clear impact of IBD‐related soluble mediators on intestinal barrier integrity by incubating PCIS culture supernatants with intestinal epithelial cells without any evidence of cell cytotoxicity. Remarkably, both IBD‐related mediator release and barrier‐disrupting effects could be inhibited by ex vivo treatment of intestinal tissue slices with the calcineurin inhibitor pimecrolimus, which primarily targets T cells. This indicates that the epithelial barrier damage observed in our study was induced mainly by steady‐state secreted T cell mediators or their downstream signaling pathways. Inhibition of calcineurin signaling by selective calcineurin inhibitors for the treatment of IBD has also been investigated by others [17, 76]. Christensen et al. [17] showed that a combination of tacrolimus or cyclosporin with vedolizumab in patients with refractory, active IBD achieves steroid‐ and calcineurin inhibitor‐free clinical remission in more than one‐third of patients after 1 year. Additionally, pimecrolimus, another calcineurin inhibitor, exhibits a selective immunomodulatory effect by inhibiting T cell activity without affecting dendritic cell differentiation or function [77], whereas tacrolimus has also been shown to affect other immune cells, including dendritic cells [78, 79]. Pimecrolimus has also been proposed as a potential therapeutic option for treating IBD and asthma and was the subject of a corresponding patent application [80]. These findings further emphasize the therapeutic relevance of targeting calcineurin signaling in intestinal inflammation. Calcineurin may therefore represent a promising therapeutic target in a subgroup of therapy‐refractory IBD patients. The significant inhibition of T‐cell‐associated cytokine release by pimecrolimus in PCIS could be attributed to the elevated T‐cell infiltration observed in the pericryptal areas of Crohn's disease patients. This high concentration of T cells likely amplifies the drug's efficacy, providing a potential explanation for its potent immunosuppressive effects in this setting.

In conclusion, our study demonstrates for the first time that primary human intestinal tissue slices are a valuable, immunocompetent model to study functional tissue‐specific responses in the context of inflammatory diseases ex vivo and to assess new therapeutic approaches. The substantial accumulation of T cells in IBD is maintained in primary intestinal tissue slices and reveals functional secretion of Th1/Th17 cytokines. Ex vivo inhibition of T cell activity via the calcineurin pathway using pimecrolimus treatment leads to a reduction of T cell‐derived cytokines after activation with ConA, providing a proof‐of‐concept for pharmacological modulation of relevant immune pathways in the system. Furthermore, pimecrolimus treatment of primary human tissue shows secondary protective effects on intestinal barrier integrity. The combination of intestinal tissue slice cultures and cell cultures provides a novel approach to better understand pathophysiological mechanisms and tissue immune responses, and test pharmaceutical interventions in a system based on human donor tissue. This system can be expanded in future studies to comprehensively assess the efficacy and mechanism of other IBD‐relevant therapeutics, such as biologics (anti‐IL‐12/23), JAK inhibitors, and other novel therapeutic approaches.

### 3.1 Data Limitations and Perspectives

In this study, we highlight the potential of human patient‐derived PCIS as a valuable model for investigating immune responses and pharmacological interventions in IBD and non‐IBD tissues ex vivo. While PCIS effectively preserve important aspects of intestinal tissue architecture and immune activation, one of its limitations is the lack of active infiltration of immune cells from circulating blood during culture; to address this limitation, future studies could include co‐cultures with autologous or peripheral immune cells to improve the model's ability to simulate infiltration of immune cells and dynamic changes in the tissue. Another limitation is the short cultivation time of 48 h, which restricts the ability to study chronic exposure and long‐term events. Integration of PCIS into a more physiological environment generated by microfluidic organ‐on‐chip systems could allow longer cultivation periods. Further, although the epithelial layer is preserved in PCIS, functional analyses of barrier integrity remain challenging and require the development of specialized assays. PCIS offers the potential to create a highly physiologically relevant, human platform for drug testing. Integrating advanced technologies will enhance the predictive value of PCIS findings and facilitate their translation into clinical trials.

## 4 Methods

### 4.1 Ethical Statement

Human intestinal tissue was obtained from patients who had undergone surgery for different indications. The experiments with human intestinal tissue were approved by the Ethics Committee of the Hannover Medical School (MHH, Hannover, Germany) and conform to the *Code of Ethics of the World Medical Association*. All patients or their relatives, caregivers, or guardians gave written informed consent for the use of intestinal tissue for research. Human tissue samples were obtained from the terminal ileum of both female and male donors (Table 1). Ileum tissue from Crohn's disease patients (IBD) and tumor‐free ileum tissue from patients with non‐IBD background diseases (non‐IBD) were used (Table 1).

### 4.2 Preparation and Cultivation of PCIS

PCIS were prepared according to an adapted protocol by de Graaf et al. [29]. Tissue resectates were rinsed and placed in oxygenated, ice‐cold Krebs–Henseleit–Buffer (KHB). The tissue was dissected free of muscle tissue and residual fat, and mucosal sheets were embedded in 3% agarose (Sigma), from which 8 mm cores were punched. Cores were cut in oxygenated, ice‐cold KHB with a microtome (Krumdieck Tissue Slicer) into approx. 300 µm‐thick tissue slices. PCIS were cultivated in 12‐well plates with two PCIS per well in 1 mL WME (with l‐glutamine, 14 mM D‐Glucose, 1X Gibco‐Antibiotic‐Antimycotic, phenol‐red) at 37°C, 95% O_2_/5% CO_2_ in a shaking incubator (90 rpm). After 1 h preincubation, the medium was exchanged, and PCIS were postincubated for 24 h with or without treatments. For stimulation of immune cells, 10 µg/mL of Concanavalin A (ConA, Sigma) or 1 µg/mL lipopolysaccharide from *E. coli O111:B4* (LPS, Sigma) were used. For inhibition of calcineurin‐NFAT signaling, 25 µM pimecrolimus (Sigma) was added, based on preliminary data showing toxicity at higher concentrations (≥50 µM). Each experiment was set up in technical duplicates or triplicates per donor (2–3 wells with two PCIS per well).

### 4.3 Cell Culture

C2BBe1 (Clone of Caco‐2, ATCC CRL‐2102) cells were purchased from American Type Culture Collection (ATCC; LGC Promochem). Cells were routinely cultivated according to ATCC culture recommendations. The cells were used at passage numbers 61–69. For the experiments, C2BBe1 were seeded on rat tail collagen type I [10 µg/mL, Thermo Fisher] coated microporous membranes with a pore size of 0.4 µm (Falcon transwells system, Corning), The cells were cultivated for 10–14 days until a dense monolayer barrier of at least 600 Ω/cm^2^ was reached, measured with EVOM Epithelial Voltohmmeter (World Precision Instruments). The medium was completely changed every 2–3 days.

For the experiments with C2BBe1 monolayer, supernatants of 24 h cultured PCIS with or without 25 µM pimecrolimus from primary intestinal tissue of IBD or non‐IBD patients were used. Cultured PCIS supernatants were purified from potential tissue debris by centrifugation (350×*g*, 3 min) and were then mixed with C2BBe1 culture medium at a ratio of 1:4 and added to the basolateral compartment of transwell cultures. As control, a 1:4 mixture of PCIS culture medium diluted in C2BBe1 culture medium was used. After 24 h of cultivation, epithelial barrier integrity and viability were assessed.

### 4.4 Histopathology

PCIS were fixed in 10% neutral buffered formalin, embedded in paraffin, and sliced into 4 µm‐thin slices. The paraffin slices were stained with hematoxylin and eosin (H&E) using standard histological procedures and mounted for light microscopy. Magnification and scale bars are indicated in the figure. For histological analyses, PCIS (cultivated for 24 h in medium) from *N* = 4 IBD and non‐IBD donors each were analyzed. Per Donor, a minimum of two replicates (two wells with two PCIS each) were processed.

### 4.5 Viability Assays

The viability of PCIS was determined either by LDH release assay from supernatants (LDH Cytotoxicity Assay Kit, Roche Diagnostics) or tissue ATP content (ATPlite luminescence assay system, PerkinElmer) according to the manufacturer's instructions. Triton X‐100 (1% in phosphate‐buffered saline (Sigma))‐treated PCIS were investigated as a reference (dead) control. For LDH measurements, multiple dilutions of the samples and controls were applied to adjust for high optical density values. Absorbance was measured at 490 and 630 nm as reference wavelengths using a microplate reader (Microplate Reader Infinite 200 Pro Tecan Group, Männedorf, Switzerland). For ATP measurements, luminescence was measured with an integration time of 2000 ms using the same microplate reader. The viability of C2BBe1 cells was determined by LDH release assays from apical supernatants and metabolic activity with the cell proliferation reagent WST‐1 (Roche Diagnostics) according to the manufacturer's instructions.

### 4.6 Analysis of Mediator Secretion

Cytokine and chemokine levels in PCIS supernatants were analyzed with a Mesoscale Discovery customized U‐PLEX assay (Mesoscale Diagnostics) according to the manufacturer's instructions. Panels included the following analytes (lower level of quantification):

IL‐1β (1.15 pg/mL), IL‐2 (0.69 pg/mL), IL‐10 (1.01 pg/mL), IFN‐γ (7.88 pg/mL), GM‐CSF (1.34 pg/mL), TNF‐α (0.78 pg/mL), IL‐4 (0.48 pg/mL), IL‐5 (1.12 pg/mL), IL‐13 (0.64 pg/mL), IL‐31 (26.45 pg/mL), IL‐17A (8.78 pg/mL), IL‐17E (1.43 pg/mL), IL‐17F (396 pg/mL), IL‐21 (15.19 pg/mL), IL‐22 (0.57 pg/mL), IL‐23 (4.51 pg/mL), granzyme A (2.63 pg/mL), granzyme B (5.83 pg/mL), IL‐18 (1.76 pg/mL), TSLP (6.28 pg/mL), IL‐33 (1.99 pg/mL), IL‐12p70 (1.39 pg/mL), MIP‐3α (5.49 pg/mL), IL‐27 (14.39 pg/mL), MCP‐1 (1.21 pg/mL), TRAIL (1.03 pg/mL), ENA‐78 (2.33 pg/mL), Eotaxin (19.78 pg/mL), Ghrelin (23.02 pg/mL), GLP‐1 (3.11 pg/mL), Leptin (57.97 pg/mL). Plates were measured with the MESO QuickPlex SQ120 (Mesoscale Diagnostics). Plotted values below the level of quantification were extrapolated by the MSD software. In addition, further analytes were measured using DuoSet ELISA kits from R&D Systems (DY206, DY208 and DY8226) according to the manufacturer's recommendations, ELISA (lower level of detection): Human IL‐6 (9.4 pg/mL), IL‐8 (31.2 pg/mL), and Calprotectin (S100A8/S100A9 Heterodimer; 93.9 pg/mL).

### 4.7 Gene Expression Analysis

For RNA isolation, PCIS from IBD and non‐IBD patients were collected in RNAlater (Invitrogen) and incubated overnight at 4°C. Afterwards, RNAlater was removed and PCIS were stored at –80°C until RNA isolation. RNA isolation was performed according to an optimized protocol by Niehof et al. [81], based on a specific homogenization and phenol extraction procedure coupled with a MagMax magnetic beads (ThermoFisher Scientific) cleaning procedure. RNA concentration (A260) and purity (A260/A280 ratio) were measured by spectrophotometry (NanoDrop 2000 Spectrophotometer, ThermoFisher). RNA integrity number (RIN) was evaluated using an Agilent 2100 Bioanalyzer (Agilent Technologies). For subsequent gene expression analysis, all samples showed RIN values >9. Transcriptome analysis of PCIS was performed using the Affymetrix GeneChip Whole Transcript (WT) PLUS Reagent Kit and the GeneChip human Clariom S Arrays according to the manufacturer's recommendations (ThermoFisher Scientific). A total of 100 ng RNA was used as starting material for target preparation. Arrays were subsequently washed, stained, and scanned using the Affymetrix GeneChip Command Console Software (ThermoFisher Scientific) with .cel files as data output.

Raw intensity data from Affymetrix Clariom S human arrays were processed using the Robust Multi‐array Average (RMA) normalization method implemented in the oligo R package. Probe annotation was performed using the pd.clariom.s.human package. For differential expression analysis, a linear model was fitted using the limma package, IBD samples against non‐IBD controls. The model design matrix included disease status as the main factor, and empirical Bayes moderation was applied to improve variance estimation. Genes with an adjusted *p*‐value < 0.05 (Benjamini–Hochberg correction) and an absolute log2 fold change (log2FC) > 1 were considered significantly differentially expressed. ORA was performed using g:Profiler to identify significantly enriched biological pathways and processes. A volcano plot was generated using the EnhancedVolcano R package to visualize differentially expressed genes. Additionally, a bubble plot was created using ggplot2 to represent enriched pathways and biological processes obtained from over‐representation analysis.

### 4.8 Whole‐Mount Immunofluorescence of PCIS

PCIS from IBD and non‐IBD patients were fixed overnight in 2% PFA and subsequently washed with PBS. PCIS from *N* = 3 IBD and non‐IBD donors, each with two PCIS per donor, were stained. The following primary antibodies were used for immunofluorescence staining: anti‐human CD3 mouse antibody (6.6 µg/mL, RUO clone, mouse IgG1 κ isotype, 344802 Biolegend), anti‐human CD4 monoclonal rabbit antibody (13.3 µg/mL ST0488 clone, rabbit IgG, MA5‐32166 Invitrogen), anti‐human CD8 monoclonal rat antibody (20 µg/mL, YTC182.20 clone, rat IgG2b isotype, MA1‐81692 Invitrogen), and anti‐human CD68 monoclonal mouse antibody (0.8 µg/mL, PG‐M1 clone, mouse IgG3 κ isotype, M0876 Dako). As secondary antibodies, a 1:400 dilution of AlexaFluor 568‐conjugated goat anti‐rabbit IgG H&L (Abcam, ab175471), AlexaFluor 647‐conjugated goat anti‐mouse IgG H+L (Jackson ImmunoResearch, 115‐605‐003), and AlexaFluor 488‐conjugated goat anti‐rat IgG H+L (Jackson ImmunoResearch, 112‐545‐003) were used. Nuclear staining was performed with 4′,6‐diamidino‐2‐phenylindole (DAPI) for 30 min, and stained PCIS were embedded in ibidi mounting medium for subsequent confocal microscopy. Z‐stacks of 20–24 µm were imaged every 1 µm with a Zeiss 800 confocal microscope using a 20× water immersion objective. The images were reconstructed in three dimensions using IMARIS 9.2.1 for the volumetric projection. Software (Bitplane).

### 4.9 Epithelial Barrier Integrity Assays

For the assessment of epithelial barrier integrity of C2BBe1 cells, TEER and fluorescein isothiocyanate (FITC)‐dextran permeability assays were performed. TEER were measured with an Epithelial Voltohmmeter (EVOM, World Precision Instruments). The measurement was performed two times per well, and two technical replicates (wells) were included for each condition. The measured values were normalized to the medium control at the start of the experiment (0 h) (set to 100%).

For the measurement of FITC‐dextran permeability after 24 h incubation with cultured PCIS supernatants, C2BBe1 cells on microporous membranes were transferred into a new culture plate with basolateral C2BBe1 culture medium. Apically, 200 µL of 4 kDA FITC‐dextran (5 mg/mL) was added, and plates were further incubated for 2 h at 37°C. Afterwards, 100 µL basolateral medium was transferred to 96‐well plates in duplicates. Fluorescence was measured spectrophotometrically (Infinite 200PRO, Tecan, Männedorf, Schweiz) (excitation: 490 nm, emission: 520 nm). Measured fluorescence values were normalized to values of cells under control conditions and displayed as *x*‐fold.

### 4.10 Statistical Analysis

Statistical analyses were performed with GraphPad Prism (version 10.1.24). An unpaired two‐tailed *t*‐test was used to compare two groups, and one‐way ANOVA with Dunnett's multiple comparisons test was used for multiple comparisons. Findings were deemed significant if *p < 0.05, **p < 0.01, ***p < 0.001, ****p < 0.0001 or ns: not significant. All data were performed as a minimum of three independent experiments.

## Author Contributions

Klaudia Grieger, Valerie Schröder, and Katherina Sewald wrote the original draft. Valerie Schröder and Klaudia Grieger performed experiments with PCIS. Klaudia Grieger performed experiments with C2BBe1 cells. Dirk Schaudien performed histopathology. Maximillian Fuchs performed bioinformatic analysis of the gene expression data. Klaudia Grieger, Valerie Schröder, Vanessa Neuhaus, Susann Dehmel, and Christina Hesse conceived and designed the ex vivo studies. Klaudia Grieger, Vanessa Neuhaus, and Susann Dehmel designed the in vitro cell culture studies. Klaudia Grieger and Valerie Schröder performed endpoint measurements, data analysis, and statistics. Armin Braun and Katherina Sewald provided resources and scientific supervision. Alexander Wagner, Ulf Kulik, Helena Linge, Benjamin Gundert, and Heiko Aselmann performed the clinical clarification of the patients and removal of the tissue. All authors reviewed and contributed to the final version of the manuscript.

## Conflicts of Interest

The authors declare no conflicts of interest.

## Peer Review

The peer review history for this article is available at https://publons.com/publon/10.1002/eji.70013.

## Supporting information




**Supporting file 1**: eji70013‐sup‐0001‐SuppMat.pdf

**Supporting file 2**: eji70013‐sup‐0002‐TableS1.xlsx

**Supporting file 3**: eji70013‐sup‐0003‐TableS2.xlsx

## Data Availability

The data that support the findings of this study are available from the corresponding author upon reasonable request.
