# Supplementary material for: Ex Vivo Modeling and Pharmacological Modulation of Tissue Immune Responses in Inflammatory Bowel Disease Using Precision‐Cut Intestinal Slices
Source: Eur J Immunol. 2025 Jul 24;55(7):e70013. doi: 10.1002/eji.70013 (PMC12288778; doi:10.1002/eji.70013)
Supplement: Supplementary file 1 — Supporting file 1: eji70013‐sup‐0001‐SuppMat.pdf [file EJI-55-e70013-s001.pdf]

Supplemental Figures

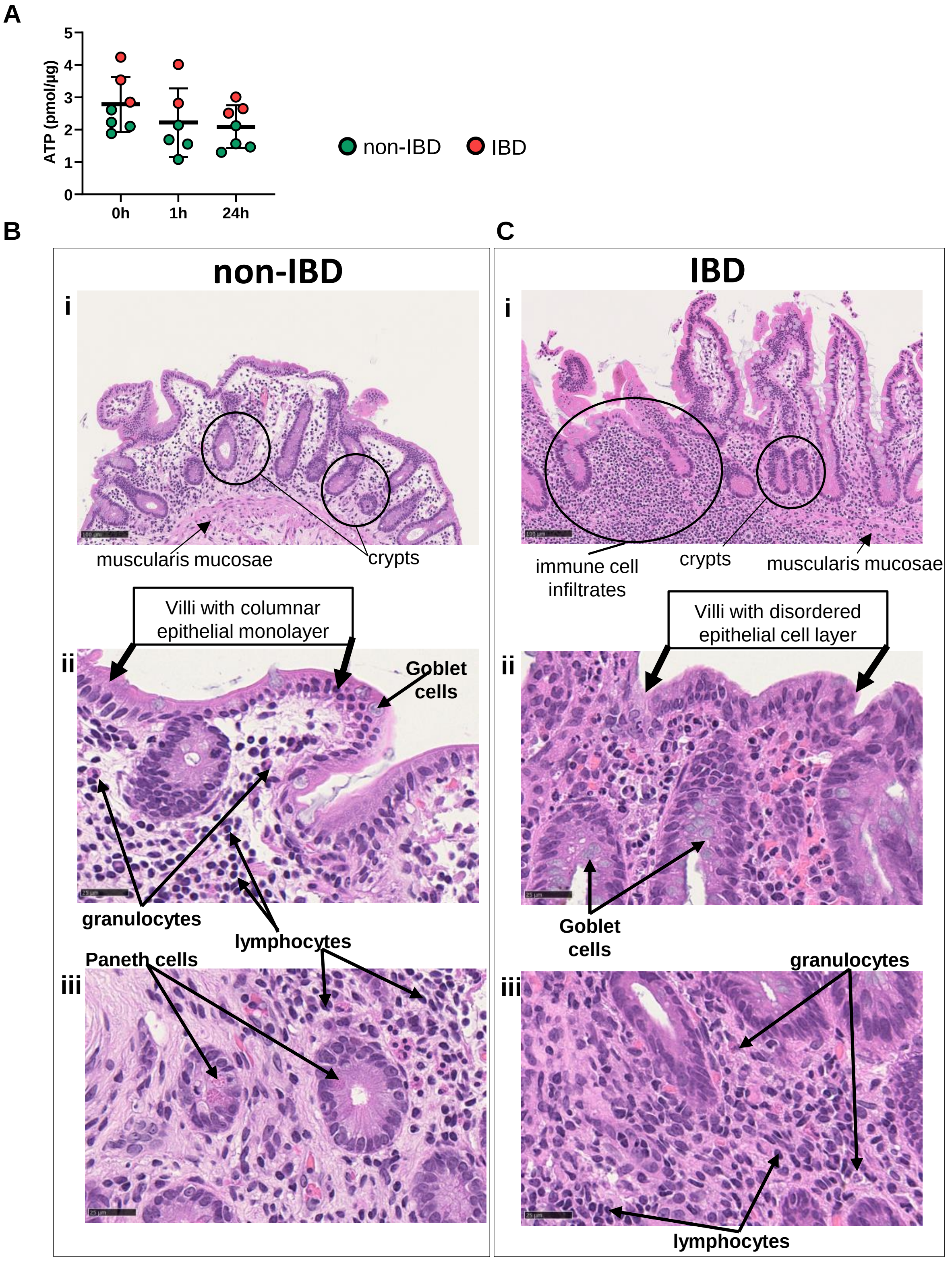

Figure S1. The ATP content and histology of intestinal tissue slices from IBD and non-IBD patients. (A) ATP content of intestinal tissue slices normalized to total protein content of intestinal tissue slices. Tissue slices were used for ATP measurement directly after cutting (0 h), after 1-hour pre-incubation phase (1 h) and after 24-hour incubation in culture medium (24 h). Each dot represents one donor, red (IBD-derived PCIS), green (non-IBD-derived PCIS), N=6-7. Per donor a minimum of two technical replicates (two wells with two tissue slices each) were analyzed. Hematoxylin & eosin staining of formalin-fixed, paraffin embedded thin sections (4 µm) of non-IBD **(B)** and IBD **(C)** tissue slices after 24 h cultivation. **(i)** 20x magnification with scale bar 100 µm and **(ii, iii)** 80x magnification with scale bar 25 µm. Circles and arrows indicate exemplary key structural and cellular features. Highlighted features: immune cell infiltrates as dense cluster of small, round basophilic nuclei, villi with columnar epithelial monolayer, crypts appear as circular/tubular structures, Goblet cells with dot-like basophilic (bluish) mucin-filled vacuoles, Paneth cells with eosinophilic (pink-red) granules, lymphocytes with deeply basophilic (dark blue-purple) round nuclei, granulocytes with segmented nuclei, muscularis mucosae with cigar-shaped muscle cells having eosinophilic cytoplasm.

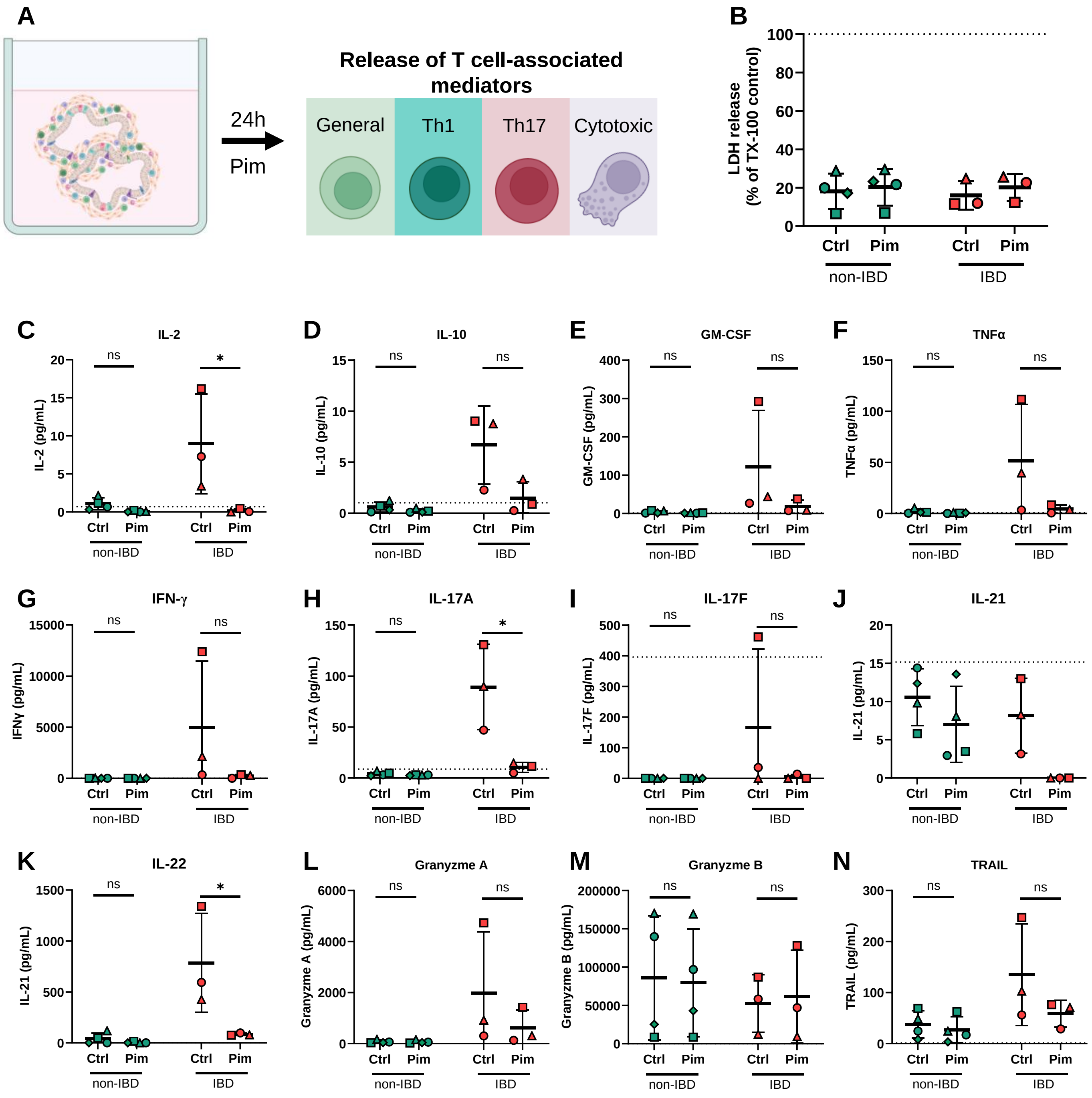

Figure S2: Pimecrolimus reduced baseline secretion of T cell-associated mediators in IBD tissue ex vivo. Tissue slices were stimulated with 25 µM pimecrolimus (Pim) for 24 h and release of T cell-associated mediators was analyzed, schematic representation **(A)**. LDH release in supernatant normalized to LDH release of Triton X-100 lysed control slices (B). IL-2 **(C)**, IL-10 **(D)**, GM-CSF **(E)**, TNF-α **(F)**, IFN-γ **(G)**, IL-17A **(H)**, IL-17F **(I)**, IL-21 **(J)**, IL-22 **(K)**, granzyme A **(L)**, granzyme B **(M)** and TRAIL **(N)** levels in supernatant. Each geometric shape represents an individual donor, non-IBD: N=4, IBD: N=3. Per donor a minimum of two technical replicates (two wells with two tissue slices each) were analyzed. *$p<0.05$, ns: not significant by unpaired two-tailed *t*-test comparing Ctrl vs. Pim. A) was generated with BioRender.com.

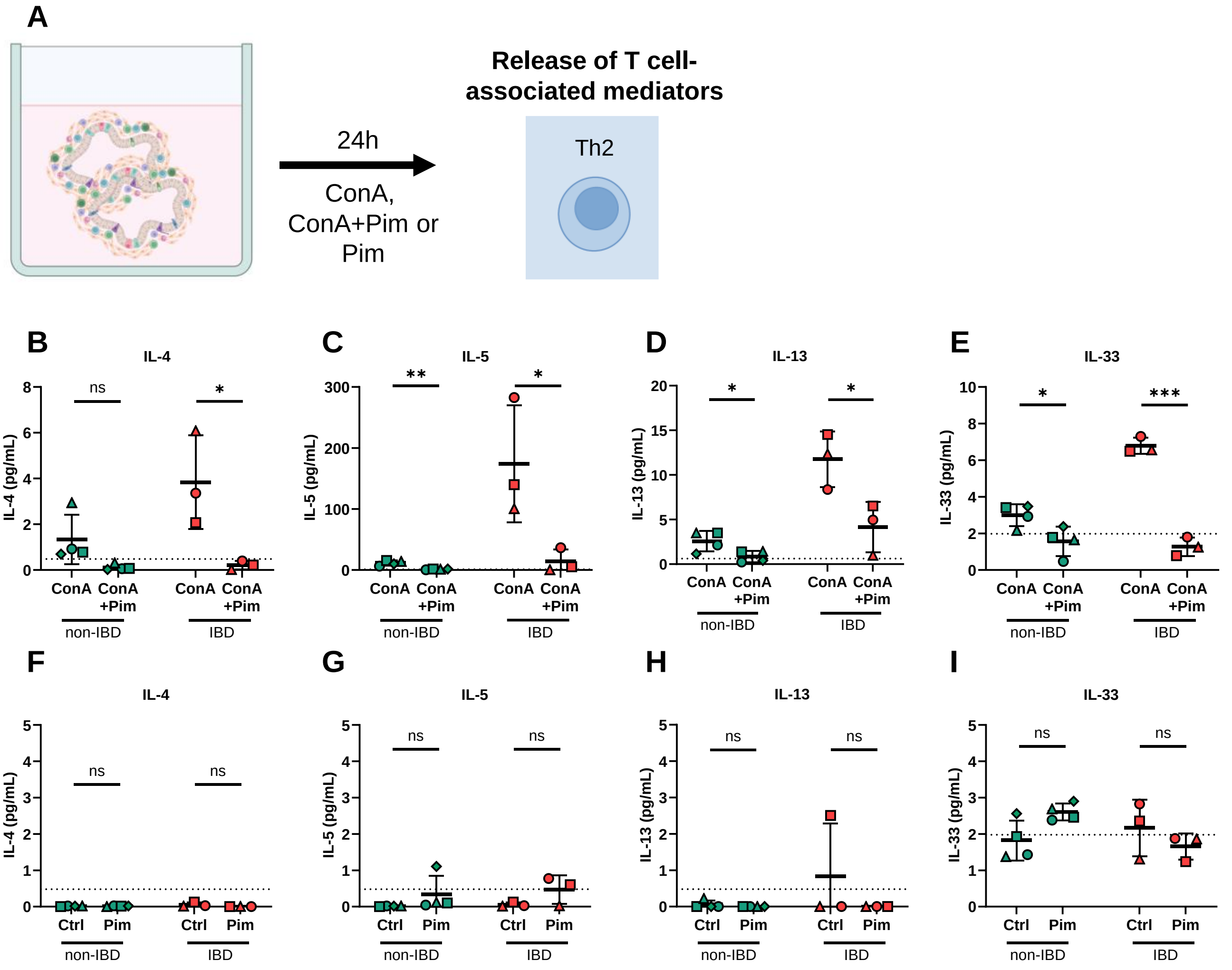

Figure S3: Release of Th2 cell-associated mediators in primary intestinal tissue slices from IBD and non-IBD patients ex vivo. Tissue slices were stimulated with Concanavalin A ± 25 µM pimecrolimus (Pim) **(B-E)** or only with 25 µM Pim **(F-I)** for 24 h and release of Th2 cell-associated mediators was analyzed, schematic representation **(A)**. IL-4 **(B, F)**, IL-5 **(C, G)**, IL-13 **(D, H)** and IL-33 **(E, I)** levels in supernatant. Each geometric shape represents an individual donor, non-IBD: N=4, IBD: N=3. Per donor a minimum of two technical replicates (two wells with two tissue slices each) were analyzed. *p<0.05, **p<0.01, ***p<0.001, ns: not significant by unpaired two-tailed *t*-test comparing ConA vs. ConA+Pim or Ctrl vs. Pim. A) was generated with BioRender.com.

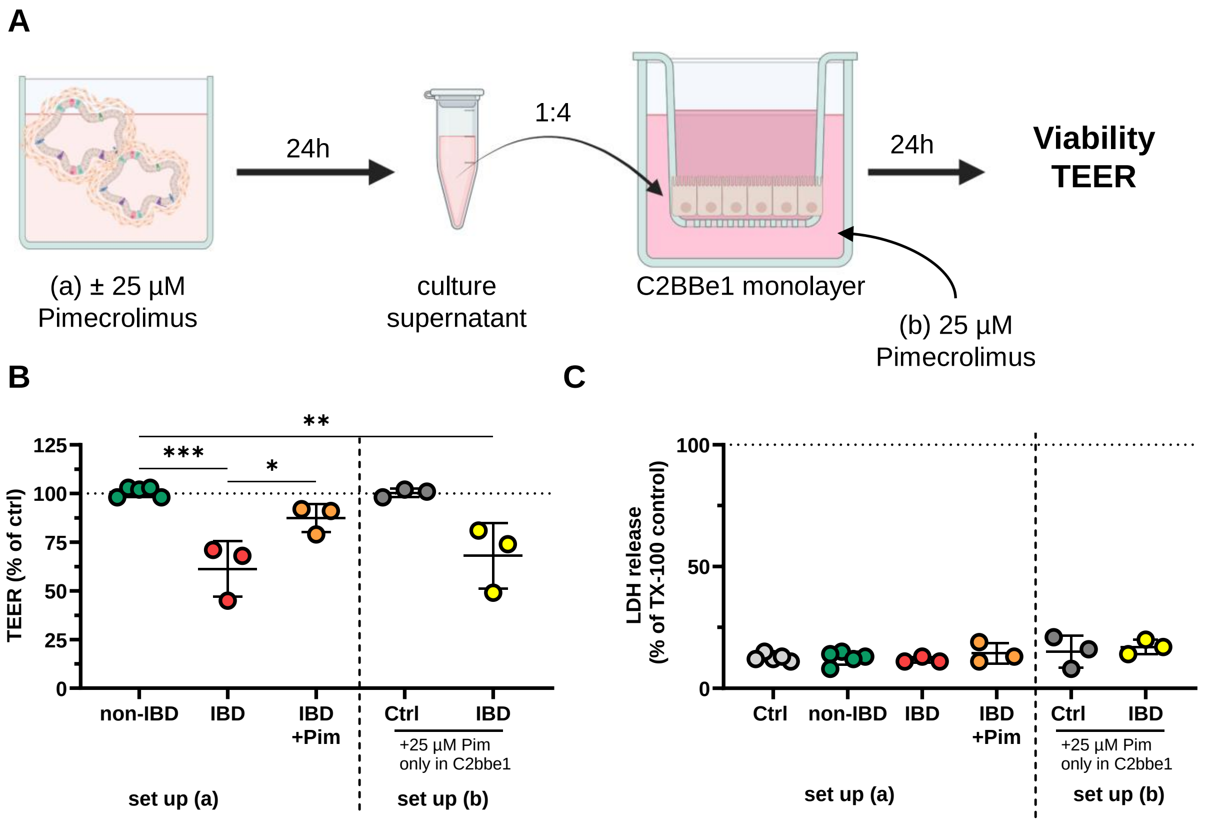

Figure S4: Pimecrolimus showed no direct influence on the C2BBe1 monolayer. Extended representation for figure 4. Supernatants of tissue from non-IBD patients, tissue from IBD patients or tissue from IBD patients treated with 25 µM pimecrolimus (Pim) were incubated on C2BBe1 monolayers for 24 h. Culture supernatants were mixed with C2BBe1 culture medium at a ratio of 1:4. A 1:4 mixture of tissue culture medium and C2BBe1 culture medium was included as medium control (Ctrl) **(A, set up a)**. For the direct effect of pimecrolimus on C2BBe1 monolayers, pimecrolimus (25 µM) was added directly to conditioned supernatants from IBD patients (IBD) or to control medium (Ctrl) and applied to C2BBe1 monolayers for 24 h **(A, set up b)**. Transepithelial electrical resistance (TEER) of C2BBe1 cells at 24 h, normalized to Ctrl at 0 h **(B)**. LDH release in apical supernatants at 24 h, normalized to Triton X-100 lysed control (**C**, dotted line, set to 100%). Each dot represents one individual donor of which supernatants for incubation on C2BBe1 cells were collected. N=5 donors (non-IBD) and N=3 donors (IBD and IBD+Pim). Per donor one experimental run with two technical replicates was performed. *p<0.05, **p<0.01, ***p<0.001 by one-way ANOVA with Dunnett's multiple comparisons test. (A) was created with BioRender.com

Table S1 Complete list of all upregulated signalling pathways in precision-cut intestinal slices from IBD patients in comparison to non-IBD patients. N=5 donors (IBD-derived PCIS) and N=4 donors (non-IBD-derived PCIS), per donor two technical replicates (two wells with two tissue slices each) were analyzed using Clariom S microarray.

Table S2 Complete list of all upregulated genes in precision-cut intestinal slices from IBD patients in comparison to non-IBD patients. N=5 donors (IBD-derived PCIS) and N=4 donors (non-IBD-derived PCIS), per donor two technical replicates (two wells with two tissue slices each) were analyzed using Clariom S microarray.

Table S3 Proinflammatory mediator levels in unstimulated and ConA-stimulated IBD and non-IBD tissue slices. Tissue slices were stimulated with 10 µg/mL Concanavalin A (ConA) or cultivated only in Medium (Ctrl) for 24 h and the absolute secretion of several mediators in supernatant was analyzed. Table depicts mean values (pg/mL) and standard deviation (SD) of N=4 non-IBD and N=3 IBD donors. Per donor a minimum of two technical replicates (two wells with two tissue slices each) were analyzed. P-values were calculated by unpaired two-tailed t-test comparing either Ctrl vs ConA or IBD vs. non-IBD.

	**non-IBD**					**IBD**						
	**Ctrl**		**ConA**		**Ctrl vs. Cona**	**Ctrl**		**ConA**		**Ctrl vs. ConA**	**IBD vs. non-IBD Ctrl**	**IBD vs. non-IBD ConA**
	**Mean [pg/mL]**	**SD**	**Mean [pg/mL]**	**SD**	**p-value**	**Mean [pg/mL]**	**SD**	**Mean [pg/mL]**	**SD**	**p-value**	**p-value**	**p-value**
**IL-1β**	2.13	2.33	21.42	6.68	**0.0033**	44.1	35.37	876.55	825.07	0.2271	0.1019	0.1402
**IL-6**	3041.37	4001.15	12868.33	4809.32	0.0905	22710.97	25809.28	63590.5	37737.35	0.2747	0.34	0.1324
**IL-8**	2873.04	975.3	53833.09	23528.2	**0.0095**	276570.01	140763.89	638078.74	369039.4	0.2652	**0.0218**	**0.0444**
**Calprotectin**	21910.26	23346.47	15876.75	19742.43	0.7442	109092	54863.42	113276.67	57031.88	0.944	**0.0499**	**0.0438**
**IL-2**	1.08	0.69	576.66	141.3	**0.0004**	8.97	5.36	1179.17	44.35	**<0,0001**	0.0572	**0.0018**
**IL-10**	0.6	0.42	41.33	38.5	0.1167	6.69	3.13	146.91	99.83	0.1181	**0.0227**	0.1634
**IFNγ**	15.29	10.54	27507.51	11528.09	**0.0061**	4955.56	5307.79	58251.3	2636.4	**0.0002**	0.1765	**0.0123**
**GM-CSF**	4.22	3.03	242.16	98.79	**0.0059**	120.93	121.19	3014.71	1851.44	0.0921	0.1646	0.464
**TNFα**	2.02	1.96	158.79	53.82	**0.0024**	51.57	44.97	1585.69	913.96	**0.0767**	0.1219	0.0528
**IL-4**	0.01	0.01	1.33	0.93	**0.0497**	0.06	0.05	3.84	1.67	**0.0333**	0.1936	0.0869
**IL-5**	0.6	0.53	11.54	3.58	**0.002**	7.75	7.37	174.29	78.29	**0.0401**	0.1633	**0.0171**
**IL-13**	0.06	0.1	2.56	0.99	**0.0047**	0.84	1.18	11.75	2.55	**0.0054**	0.3168	**0.0026**
**IL-31**	65.74	7.05	73.19	4.45	0.1724	22.59	17.38	84.33	5.51	**0.0087**	**0.0126**	0.0544
**IL-17A**	4.28	1.76	1181.06	806.57	**0.0449**	89.23	34.16	8064.49	5083.15	0.0907	**0.0085**	0.0371
**IL-17E**	0.25	0.34	1.82	0.23	**0.0006**	0.96	0.46	5.36	0.54	**0.001**	0.1068	**0.0002**
**IL-17F**	*26,9#*		203.13	107.7	**0.0171**	153.92	217.68	1979.67	239.44	**0.0013**	0.2856	**0.0001**
**IL-21**	10.59	3.21	14.76	1.94	0.1024	8.15	4.01	36.77	9.68	**0.0181**	**0.4834**	**0.0134**
**IL-22**	41.56	48.61	2807.51	606.9	**0.0002**	785.76	397.39	7198.77	250.89	**<0,0001**	0.0258	**0.0002**
**IL-23**	2.17	3.75	21.93	20.43	0.1505	4.97	2.19	61.94	8.7	**0.0009**	0.3738	0.0433
**GzmA**	75.18	55.13	7288.51	2742.41	**0.0039**	1983.92	1958.54	12323.16	323.85	**0.0018**	0.1606	0.0442
**GzmB**	85995.65	70187.75	57558.22	45084.6	0.5764	52550.83	30679.72	37570.96	19322.27	0.5904	0.543	0.5695
**IL-18**	3.13	2.7	10.03	4.85	0.0746	25.9	11.76	133.69	133.58	0.3191	**0.025**	0.1787
**TSLP**	41.42	20.22	81.36	27.09	0.0867	199.6	174.25	503.54	316.62	0.3001	0.1888	0.0749
**IL-33**	1.82	0.48	2.99	0.52	**0.0288**	2.16	0.63	6.78	0.36	**0.0009**	0.5223	**0.0003**
**IL-12p70**	0.05	0.08	1.8	0.74	**0.0067**	0.74	0.18	9.14	1.46	**0.0013**	**0.0025**	**0.0007**
**MIP3α**	17.66	11.77	376.51	278.49	0.0673	39.64	26.36	102.41	19.44	0.535	0.2656	0.2099
**IL-27**	16.38	12.76	10.62	6.86	0.5168	9.46	11.9	58.37	13.74	**0.019**	0.56	**0.0038**
**MCP-1**	1752.48	1028.74	4924.95	221.34	**0.002**	4195.33	1080.78	5291.43	146.98	0.2283	**0.05**	0.0896
**TRAIL**	37.59	23.13	298.79	151.84	**0.0258**	135.39	81.31	366.21	32.49	**0.0203**	0.1113	0.5509
**ENA-78**	206.7	276.65	2050.17	1651.52	0.1052	3977.55	1418.18	4907.68	93.38	0.4071	**0.0071**	0.0525
**Eotaxin-1**	21.75	9.94	43.35	13.89	0.071	42.25	9.49	72.67	8.63	**0.0285**	0.0675	0.0417
**Ghrelin (total form)**	5.48	9.49	33.56	22.59	0.0944	10.36	14.66	41.85	2.46	**0.0401**	0.6709	0.6155
**GLP-1 (totale form)**	2.38	1.35	5.94	3.54	0.1545	52.32	40.67	28.07	22.34	0.501	0.0928	0.1605
**Leptin (totale form)**	7.31	12.66	89.97	85.58	0.149	45.42	31	198.93	48.44	**0.0195**	0.119	0.1551

Table S4 Proinflammatory mediator levels in LPS-stimulated IBD and non-IBD tissue slices. Tissue slices were stimulated with 1 µg/mL lipopolysaccharide (LPS) for 24 h and the absolute secretion of several mediators in supernatant was analyzed. Table depicts mean values (pg/mL) and standard deviation (SD) of N=3 non-IBD and N=3 IBD donors. Per donor a minimum of two technical replicates (two wells with two tissue slices each) were analyzed. P-values were calculated by unpaired two-tailed t-test comparing either Ctrl vs LPS or IBD vs. non-IBD.

	non-IBD					IBD					
	Ctrl		LPS		Ctrl vs. LPS	Ctrl		LPS		Ctrl vs. LPS	IBD vs. non-IBD LPS
	Mean	SD	Mean	SD	p-value	Mean	SD	Mean	SD	p-value	p-value
**IL-1β**	0.96	0.29	14.09	4.65	**0.016**	44.10	35.37	119.69	92.91	0.340	0.184
**IL-6**	165.15	80.29	4864.01	5237.05	0.273	22710.97	25809.28	33466.00	25602.65	0.697	0.197
**IL-8**	2660.34	1050.62	29831.57	15849.30	0.073	276570.01	140763.89	213500.71	108191.02	0.642	0.076
**Calprotectin**	8965.87	9364.06	3760.73	1503.58	0.481	109092.00	54863.42	161120.67	81876.74	0.497	0.053
**IL-2**	1.17	0.96	4.68	5.08	0.391	8.97	5.36	3.14	2.04	0.224	0.712
**IL-10**	0.30	0.13	7.59	5.63	0.141	6.69	3.13	47.29	19.44	0.043	0.050
**IFNγ**	8.83	1.57	150.48	118.89	0.167	4955.56	5307.79	10823.35	13015.89	0.587	0.311
**GM-CSF**	1.31	0.20	18.26	9.67	0.068	120.93	121.19	218.14	217.07	0.061	0.263
**TNFα**	0.69	0.35	12.87	4.26	**0.016**	51.57	44.97	281.29	327.13	0.381	0.311
**IL-4**	0.01	0.01	0.02	0.01	0.531	0.06	0.05	0.02	0.03	0.392	0.999
**IL-5**	0.66	0.55	1.19	1.21	0.602	7.75	7.37	3.56	2.62	0.491	0.311
**IL-13**	0.00	0.00	0.21	0.17	0.149	0.84	1.18	0.75	0.59	0.930	0.284
**IL-31**	67.60	8.65	71.38	2.03	0.580	22.59	17.38	45.87	6.16	0.149	0.051
**IL-17A**	6.26	5.05	22.94	16.15	0.237	89.23	34.16	72.50	21.30	0.588	0.059
**IL-17E**	0.81	0.66	1.06	0.36	0.660	0.96	0.46	1.09	0.03	0.710	0.919
**IL-17F**	0.00	0.00	0.00	0.00	0.999	153.92	217.68	109.75	155.21	0.827	0.374
**IL-21**	11.18	3.22	17.33	0.45	0.056	8.15	4.01	10.16	3.84	0.636	0.059
**IL-22**	1.35	1.82	374.78	260.03	0.112	785.76	397.39	1638.96	497.88	0.131	**0.033**
**IL-23**	0.00	0.00	42.67	28.72	0.140	4.97	2.19	213.74	242.20	0.290	0.344
**GzmA**	49.56	10.26	178.47	93.02	0.123	1983.92	1958.54	3600.02	3320.51	0.585	0.219
**GzmB**	60736.18	55970.36	57044.40	47482.97	0.947	52550.83	30679.72	59524.67	27237.89	0.822	0.952
**IL-18**	1.99	1.21	4.05	1.56	0.215	25.90	11.76	21.87	7.90	0.708	0.035
**TSLP**	19.89	3.24	66.64	28.31	0.081	199.60	174.25	276.38	219.78	0.718	0.252
**IL-33**	2.18	0.53	1.98	0.59	0.738	2.16	0.63	1.43	0.16	0.185	0.264
**IL-12p70**	0.06	0.09	0.11	0.01	0.430	0.74	0.18	1.15	1.23	0.666	0.299
**MIP3α**	18.41	9.77	114.20	117.57	0.315	39.64	26.36	55.64	34.55	0.630	0.536
**IL-27**	12.63	11.50	33.41	9.05	0.115	9.46	11.90	33.15	10.66	0.104	0.980
**MCP-1**	902.67	161.84	2832.93	1316.56	0.109	4195.33	1080.78	4695.69	477.45	0.582	0.133
**TRAIL**	28.26	18.10	49.56	29.06	0.429	135.39	81.31	178.79	52.00	0.559	**0.037**
**ENA-78**	67.96	77.20	1312.63	995.00	0.153	3977.55	1418.18	4636.28	253.23	0.553	**0.010**
**Eotaxin-1**	15.97	6.45	14.14	10.42	0.477	42.25	9.49	38.60	9.78	0.724	0.073
**Ghrelin (total form)**	0.13	0.19	3.16	3.55	0.166	10.36	14.66	17.66	16.26	0.398	0.285
**GLP-1 (totale Form)**	4.29	4.24	2.43	2.08	0.609	52.32	40.67	24.64	18.85	0.432	0.173
**Leptin (Totale Form)**	3.03	4.28	0.00	0.00	0.374	45.42	31.00	17.88	25.29	0.385	0.374
